# Gene driven analytical learning model for accurate breast cancer diagnosis

**DOI:** 10.1038/s41598-026-39430-6

**Published:** 2026-03-03

**Authors:** Farah Hesham, Mohamed M. Abbassy, Mohammed Abdalla

**Affiliations:** 1 Information Technology Program,The Egyptian-Korean Faculty of Technological Industry and Energy, Beni-Suef Technological University (BTU), Beni-Suef, Egypt; 2 Faculty of Computers and Artificial Intelligence, Beni-Suef University, Beni-Suef, Egypt

**Keywords:** Breast cancer prognosis, Hybrid CNN-BiLSTM, Gene-gene interaction, Transcriptomic biomarkers, External validation, Hyperparameter tuning, Cancer, Computational biology and bioinformatics

## Abstract

Patients diagnosed with breast cancer exhibit a diverse range of prognostic outcomes due to the varied nature of the disease across different patient groups. To address this complexity and enhance prognostic predictions based on gene expression data from breast cancer samples, this study has developed an integrated deep learning method that combines Convolutional Neural Networks (CNN) with Bidirectional Long Short-Term Memory (BiLSTM) networks. This automated pipeline conducts a correlation analysis using Pearson correlation to derive a reliable 236-gene set, ensuring no data contamination from patient samples.Furthermore, patterns of gene–gene interactions based on correlations were examined to provide further evidence of the biological relevance of the gene set that was selected. The training and validation of the proposed model utilized data from The Cancer Genome Atlas-Breast Cancer (TCGA-BRCA) and was assessed using the METABRIC dataset to enhance generalization capabilities. Experimental results indicate that the Full Hybrid (CNN BiLSTM) model significantly outperforms other machine learning and deep learning approaches. Notably, while the BiLSTM-only model achieved an optimal Recall of 0.9319, the hybrid model demonstrated a substantially higher Recall of 0.9943, accompanied by an impressive ROC AUC of 0.9955 and an F1 score of 0.9962. Furthermore, the proposed framework has been statistically validated, achieving a minimal variance of 0.000083 even under conditions of up to 20% noise perturbation. Optimization of this framework was conducted using the Optuna Bayesian Optimization methodology on a dual NVIDIA Tesla T4 array configuration. Overall, this article presents a universal computational tool for precision medicine in breast cancer, designed to yield consistent results across diverse patient scenarios.

## Introduction

**Fig. 1:**
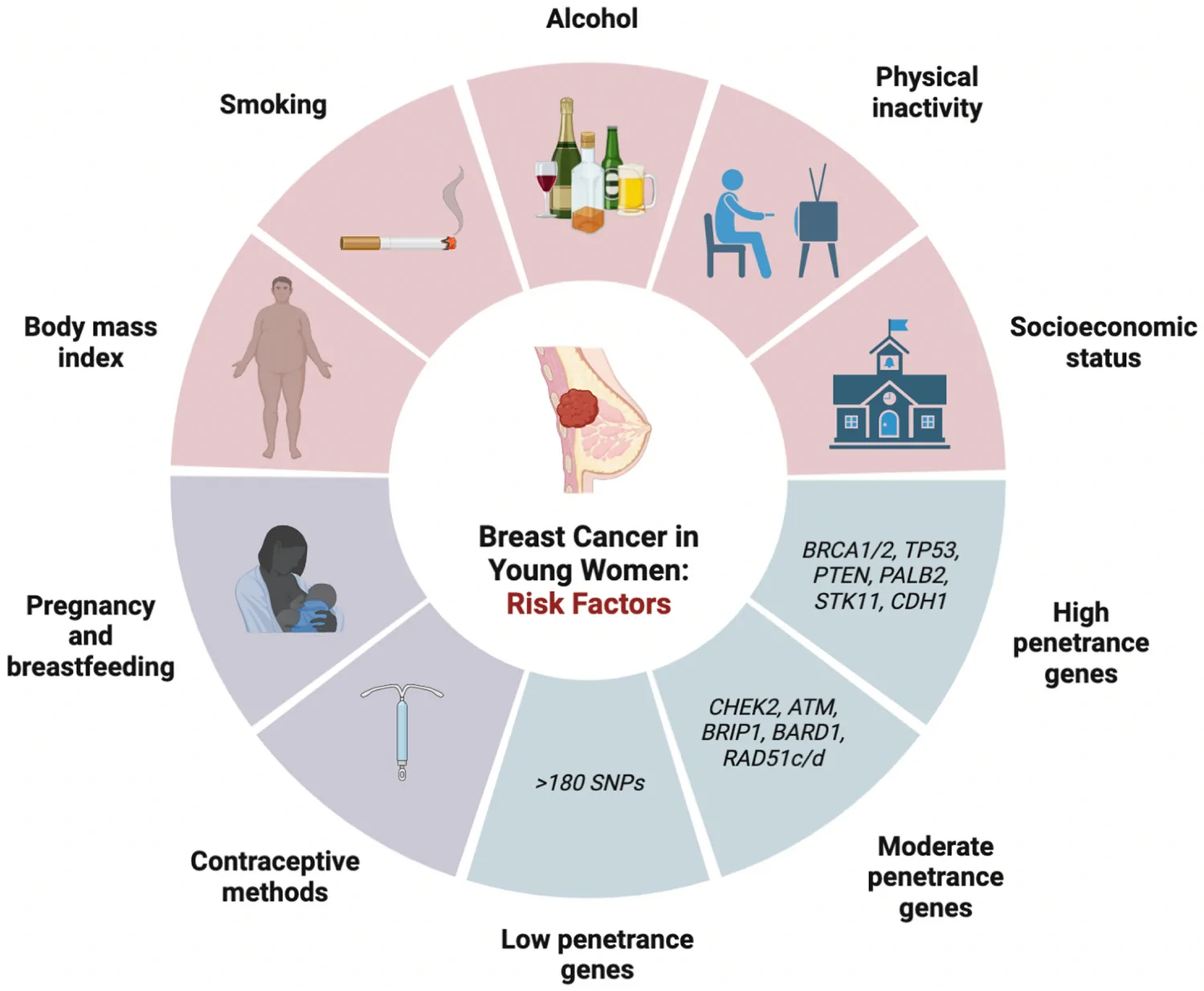
The multifactorial risk factors associated with breast cancer development.

It has been documented that breast cancer is now the most common form of cancer diagnosed among women, having surpassed lung cancer as the most diagnosed form of all cancers in the past year^1^. The raw data show an alarming rise in breast cancer diagnoses, with approximately 2.3 million newly diagnosed cases (approximately 11.7%) of all cancer diagnoses in 2020 and a staggering number of deaths due to breast cancer estimated at 684,996^2^. In developing countries, the incidence rate and mortality rate for breast cancer are increasing at alarming rates^3^. Those with genetic mutations found on BRCA1, BRCA2, TP53, and PTEN genes have a much higher likelihood of being diagnosed with breast cancer, due to the increased risk conferred by these specific genetic mutations^4^. Other factors that alter a person’s overall susceptibility include age, hormone levels, body weight, and radiation exposure^5^. Figure 1 illustrates the multifactorial risk factors associated with BC.

Recent studies demonstrate how deep learning algorithms can identify cancer with more accuracy based on genetic data that has a high degree of variation^6^. However, a major challenge in this area of study is defining the important genes in addition to dealing with the imbalance of the different classes found in genetic datasets^7,8^.In recent years, new research has identified ways in which optimization approaches to enhancing deep learning efficacy may provide a viable alternative to adding complexity to existing models in order to reduce the likelihood of overfitting, as well as reduce the amount of computational resources needed for small biomedical data sets^9^. As a result, most of the studies published so far have either utilized or are based on classical machine learning or traditional deep learning techniques. These techniques have difficulty accurately representing the spatial/temporal relationships between genes^10^. Methods that are directed towards generating efficient deep learning algorithms around improving diagnostic decisions have been presented as an effective means of eliminating over-fit and high computational costs while preserving classification performance^11^. Furthermore, the way genes are analyzed in most studies is by treating them as individual features without accounting for the complex partial correlations relationships and interactions that take place among many genes during the onset of cancer.The focus of recent research into recent advances in deep learning framework design, is creating clinical interpretability for the features represented as well as making sure that those representations are robust and consistent across a wide range of datasets^12^. Some research has been focused on a different view of the hybrid layer; however, the ultimate aim of the current research was to create an optimal pipeline with a leak free pipe between the biological interpretation of the combination of genes and the prediction of the model, filling in the gaps that have been previously identified. A new hybrid model will be presented, using the hybridization of a Convolutional Neural Network (CNN) and a Bi-directional Long Short Term Memory network (Bi-LSTM). The proposed hybrid model incorporates not only the biological significance of the correlation-based selection of 236 genes, but it also includes the discovery of gene interactions during the Optuna Bayesian Optimization process. The current research differs from other recent work in that the hybrid model will be constructed and validated using The Cancer Genome Atlas-Breast Cancer (TCGA-BRCA).

The primary contributions of this research, structured according to the experimental workflow and quantitative performance gains, are as follows:

- *Data imbalance mitigation* Implementation of a class-weighting strategy to address class imbalance in the TCGA and METABRIC datasets. By deliberately excluding synthetic data generation methods like SMOTE, we ensure that the diagnostic results are based entirely on authentic biological samples, preserving the natural integrity of the genomic data.
- *Leakage-free feature selection* Development of a rigorous pipeline that integrates Pearson correlation-based ranking within the cross-validation loop, identifying a stable 236-gene signature while effectively eliminating data leakage and ensuring reproducibility.
- *Gene interaction mapping* Providing an explicit gene correlation and interaction analysis that reveals the synergistic patterns among the identified biomarkers, justifying both the study title and the hierarchical design of the hybrid model.
- *Hybrid architecture superiority* Design of a novel CNN-BiLSTM model that achieved a significant performance leap, reaching a mean recall of 0.9943. This represents a substantial improvement of approximately 6.24% over the standalone BiLSTM model, which achieved a recall of 0.9319.
- *Bayesian hyperparameter optimization* Employment of the Optuna framework to fine-tune architectural parameters, resulting in a near-perfect ROC-AUC of 0.9955 and achieving a remarkably low variance of 0.000083, which indicates exceptional statistical stability.
- *Cross-cohort validation and robustness* Systematic evaluation of the model’s generalizability through external validation on the METABRIC dataset where the model maintained an ROC-AUC above 0.985.

The remainder of this paper is structured as follows: Section 2 reviews related works. Section 3 details the materials and methodology. Section 4 discusses the experimental results, and Section 5 presents the conclusions.

## Related work

Some early studies in computer-aided diagnosis (CAD) for breast cancer have explored various data modalities, including clinical, image-based, and high-dimensional genomic profiles. This section provides a comprehensive overview by categorizing the studies based on their data sources and methodologies. While the focus predominantly lies on genomic and transcriptomic data, numerous investigations have utilized a mix of data types such as clinical information, imaging data, and histopathological data. These aspects are examined in detail in separate reviews, highlighting their contributions to CAD systems and the differentiation between image-based and genomic predictive models.

### Clinical and image-based diagnostic models

This type of research usually involves structured clinical data or imaging, for instance, mammograms, that can help increase the accuracy of diagnoses In^13^, the researchers built a CAD system using seven machine learning models, comprising both SVM and XGBoost models and an ensemble of both models using stacking techniques. Its performance on the Mammographic Mass dataset gave an XGBoost model Recall of 96% and an ensemble model Recall of 95.35% on the Wisconsin dataset.

In^14^, a hybrid metaheuristic technique, named hGSAEPO, was designed and implemented by the authors on the Wisconsin Diagnostic Breast Cancer dataset. The technique reported an impressive precision value of 0.9800 and a Recall(sensitivity) value of 0.9700, emphasizing the role of optimal subsets of features in enhancing the accuracy of the diagnosis system.

In^15^, they offered a diagnostic scheme based on meta-heuristic optimization techniques like Genetic Algorithm (GA) and Chemical Reaction Optimization (CRO) combined with SVM, Random Forest, or XGBoost classifiers. This paper adopts three clinical datasets: Wisconsin Breast Cancer (WBC), BC-UCI, and Breast Cancer Coimbra (BCC) to evaluate their scheme. The result of feature optimization done by them showed remarkable improvement in accuracy and achieved Precision & Recall of 99.64% on WBC and 98% on the BCC dataset, respectively. This paper highlights how GA and CRO can play an important role in discovering effective clinical features for early-stage tumors.

In^16^, nomograms were created to predict 3-, 5-, and 10-year OS and BCSS in YBC patients. With 21,753 cases in the SEER dataset, they selected ten prognostic predictors using Cox modeling. The nomograms showed high prediction power, with C-indexes of 0.806 and 0.813 for OS and BCSS, respectively, in the calibration curve in the training dataset. The clinical utility of these nomograms has been validated with an independent dataset, namely METABRIC, as an external validation cohort to create a risk stratification platform to distinguish between high-risk and low-risk groups.

In^17^, an AI framework was created that predicted the histologic grades of patients from&E-stained wholeSlide scans provided in TCGA. It utilized UNI’s pathology foundation model and 14 individual multiple instance learning methods. Their work had an F1 score of 0.731 and an average AUC of 0.835 for their multiclass model. Moreover, they showed correlations between model predictions and an associated top 300 gene for cell division that supported the role of image-derived characteristics in predicting overall survival at 5 years.

In^18^, the authors developed a deep learning model based on DenseNet-161 architecture to classify breast cancer histopathological images into eight subtypes (four benign and four malignant types) using the BreakHis dataset. The proposed model employed the One Fit Cycle Policy with Cyclical Learning Rates for efficient training, achieving 97.7% accuracy at image-level and 98.68% at patient-level on 40X magnification with augmented data. This approach significantly outperformed baseline methods while reducing training time by using only 55-90 epochs compared to 300 epochs in previous studie

### Genomic and transcriptomic-based models

In^19^, the role of mitochondrial inner membrane protein (IMMT) as a molecular biomarker in breast cancer was analyzed using The Cancer GenomeAtlas (TCGA) and clinical samples. Their results confirmed that IMMT expression was highly overexpressed in breast cancer tissue relative to normal tissue. In their work, they discovered IMMT to be an independent prognosis factor that positively correlates with clinical factors like tumor size (*>2*cm), Ki-67 expression (*>15%*), and HER-2 status. Moreover, they employed Gene Set Enrichment Analysis (GSEA) to identify that IMMT affects cancer development by modulating 16 metabolism-related genes, establishing its value in metabolism-targeted therapies.

In^20^, The research analyzed 86 HER2-positive breast cancer biopsies using RNA-seq to identify recall markers for neoadjuvant therapy. DUSP4 was identified as a crucial protein that enhances therapy recall by inhibiting the ROS pathway. Its high expression is an independent predictor of positive outcomes for both Disease-Free Survival (DFS) and Overall Survival (OS). Additionally, functional analyses revealed that DUSP4 suppresses G6PD function, underscoring its role as a significant prognostic indicator of therapeutic recall in HER2 breast cancer subtypes.

In^21^, The expression pattern of MET, ESR1, and ESR2 genes was explored using the METABRIC database for determining their relationship with clinicopathologic properties. Analysis showed that MET mRNA expression is inversely related to ESR1 expression (r = −0.379) and directly associated with ESR2. Lack of significant difference in overall survival was seen among the whole cohort, but when divided according to different treatments like chemotherapy and hormonal therapies, overall survival differences were seen according to MET and ESR coexpression. These data implicate a potential molecular interaction between MET and ESR, making these genes potential new prognosticators for combined-target therapies.

In^22^, a risk signature consisting of 10 genes (CDK19 and MAP2K1 included) was established based on microarray data and LASSO regression analysis for predicting the risk of lung metastases in patients with breast cancer. The predictive classifier, established through logistic regression analysis, showed high predictive power with an Area Under the Curve (AUC) of greater than 0.87 for both the training and validation sets and was also applied to the METABRIC database with an AUC of 0.706. Both Kaplan-Meier and Cox regression analyses showed that this genomic biomarker is an independent indicator of reduced lung metastasis-free survival and overall survival, mainly within early-stage patients.

In^23^, a hybrid deep learning model integrating Bi-directional Long Short-Term Memory and Convolutional Neural Network models was proposed for survival predictions based on multi-omics data in the METABRIC and TCGA-BRCA cancer datasets. For improved interpretability and efficacy, the paper proposed the integration of minimum redundancy and maximum relevance as the method for feature selection. The results indicate substantial improvements, as the proposed model posted accuracy rates of 98% on the METABRIC and 96% on the TCGA datasets. It is safe to conclude that the combination of CNN and BiLSTM methodologies is effective in extracting the inherent spatial and temporal information in the genomic and clinical data modalities.

Despite progress by the above-mentioned studies, several limitations persist. Most genomic-based methods rely on small predefined gene sets, apply feature selection outside the cross-validation loop, or do not provide a biological rationale for sequential modeling. Another aspect that has received limited attention so far is model stability and variance between folds. Motivated by these gaps, the present study proposes a leakage-free, biologically grounded hybrid CNN-BiLSTM framework with prospective validation and stability analysis.

## Methodology

The presented approach is a well-organized workflow designed to handle dense data dimensions in genome scale as such for accurate prognosis class splitting. The overall process is divided into four major parts: data collection, feature selection based on gene correlation, design of the hybrid model architecture, and hyperparameter tuning.

### Dataset acquisition and source integrity

The BC-TCGA dataset (Breast Cancer—The Cancer Genome Atlas) is a publicly available dataset accessible via the Kaggle repository, allowing for transparency and reproducibility of analysis^24^. The Kaggle repository provides a curated mirror of the original TCGA-BRCA gene expression data without additional preprocessing, ensuring consistency with the primary TCGA source. The dataset is of large dimensionality (17,815 genes for each sample), representing a large transcriptomic space, and the data is presented as raw, unfiltered, and without the influence of external factors. This allows the model to take into account the entirety of the dataset’s genetic basis for breast cancer, rather than removing genes based only on some form of dimensionality reduction. This study utilized 590 clinical samples that represented the normal class distribution: 529 cancer and 61 normal. Given the normal class distribution had an approximate ratio of 8.6 to 1, a cost-sensitive learning method was applied. The class weights for the cost-sensitive learning method were calculated with the following formula, which is inversely proportional to the sample frequency for each class^25^. When training the model, more emphasis (weight) was given to the minority class. This ensured that the training model remained consistent with the natural (real-life) gene distribution found within the dataset and did not create any artificial weighting of the samples and their associated genes. This study calculated that there are a total of 17,815 unique genes present in the dataset. No artificial noise or data perturbation was applied to the gene expression data, as such modifications may introduce biologically implausible values and distort genuine gene–gene relationships.

### Leakage-free feature selection and correlation analysis

To identify an accurate gene signature from the first 17,815 features, we designed a multistep leakage-free pipeline that used stratified 5-fold cross-validation as a backbone. Within each training phase of this process, we used the Pearson correlation coefficient (*r*) to determine a gene’s feature ranking by determining the degree of direct linear correlation of the gene expression level with a specific clinical characteristic^26^. The formula for calculating the Pearson correlation coefficient for a particular gene is:

1 $$\begin{aligned} r = \frac{\sum _{i=1}^{n} (G_i - \bar{G})(y_i - \bar{y})}{\sqrt{\sum _{i=1}^{n} (G_i - \bar{G})^2 \sum _{i=1}^{n} (y_i - \bar{y})^2}} \end{aligned}$$

Where *n* represents the total number of samples within the training fold, $G_i$ denotes the expression value of a specific gene, $y_i$ is the target class label, and $\bar{G}, \bar{y}$ are the respective sample means.

The choice of the top 300 genes (approximately 1.7% of the total features) in each training fold was made to strike a balance between the biological significance and the efficiency of processing. The choice of this criterion was to bypass the information bottleneck that would be created by considering much smaller sets while filtering out the noise contained in the remaining 98.3% of the transcriptome. Further processing was carried out by the StandardScaler, which was fitted only on the chosen features of the training fold and used on the validation fold to ensure there was no information leakage^27^.

A stable set of 236 genes was identified as the final gene signature by taking the intersection of all the gene lists from the top-ranked genes in the first five folds and confirming those results with other populations to make sure the biomarkers had a level of reliability across numerous patient populations

**Fig. 2:**
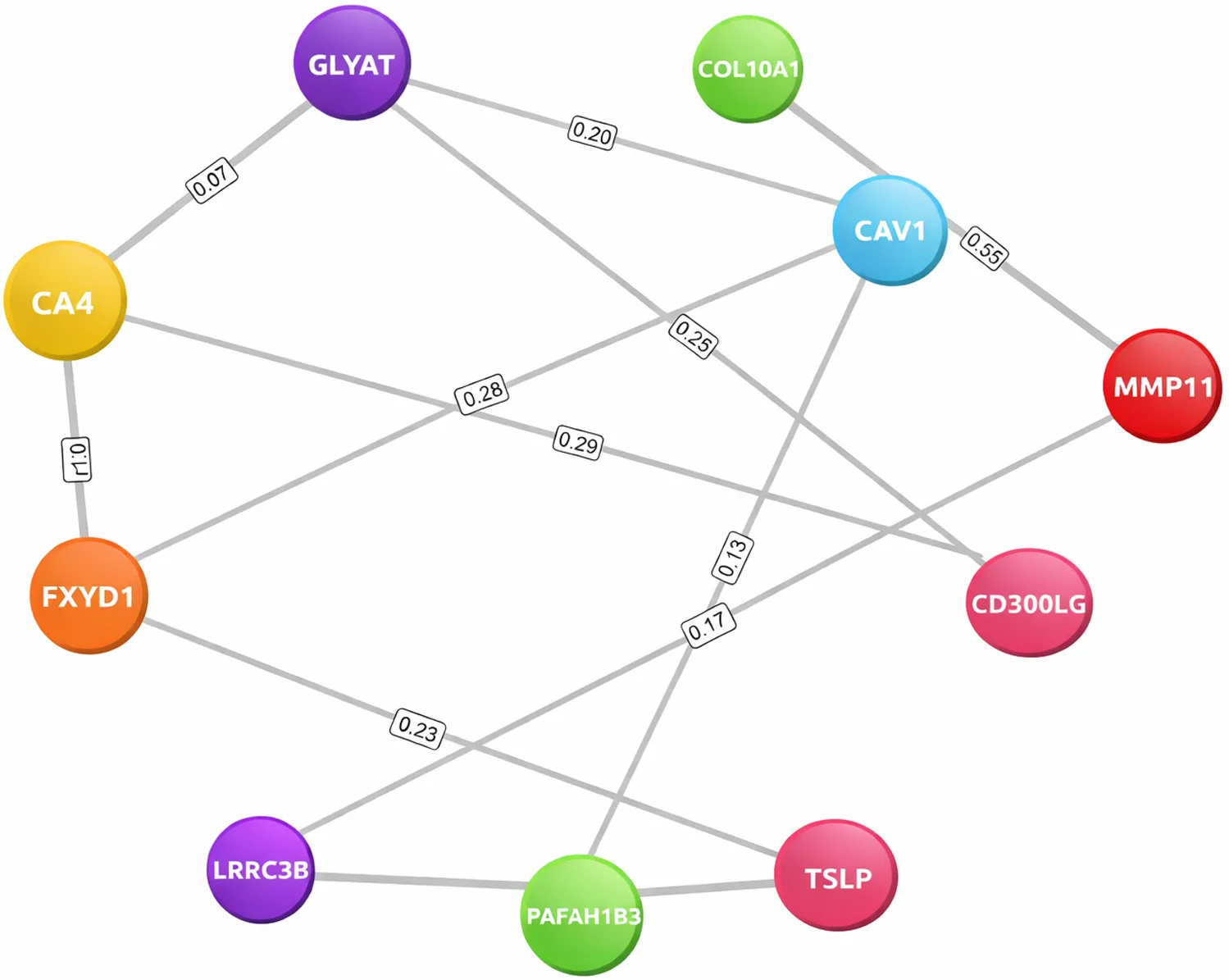
Gene–gene interaction network of the top 10 biomarkers derived from the stable gene signature. Edges represent conditional associations inferred using Graphical Lasso after standardization. Edges were filtered using an absolute partial correlation threshold ($|\rho | \ge 0.15$) and top-K filtering to avoid dense connectivity.

As illustrated in the Gene–Gene Interaction (GGI) network in Fig. 2, the selected biomarkers exhibit notable conditional dependencies (partial correlations), indicating coordinated biological behavior after controlling for the remaining genes in the network. The functional roles, documented interaction partners, and clinical relevance of these biomarkers are summarized in Table 1. Together, this interactome mapping and the supporting biological evidence provide a clear rationale for employing a hybrid architecture capable of modeling complex, non-linear dependencies among these genes.

**Table 1:** Genes risk matrix consistent with the partial-correlation network shown in Fig. 2.

Ref.	Gene	Functional role	Associated genes	Clinical risk relevance
^28^	CA4	pH regulation and tumor microenvironment	GLYAT, FXYD1, CD300LG	Tumor hypoxia adaptation
^29^	CAV1	Cell signaling regulator and tumor suppressor	FXYD1, GLYAT, PAFAH1B3	Progression and metastasis
^30^	MMP11	Extracellular matrix remodeling	COL10A1, LRRC3B	Invasion and poor prognosis
^31^	COL10A1	Matrix structural protein	MMP11	Aggressive tumor marker
^32^	TSLP	Immune modulation cytokine	FXYD1, LRRC3B	Tumor immune response
^33^	CD300LG	Immune cell adhesion molecule	CA4, GLYAT	Immune infiltration patterns
^34^	PAFAH1B3	Lipid metabolism signaling	CAV1	Metabolic reprogramming
^35^	FXYD1	Ion transport and stress response	CA4, CAV1, TSLP	Subtype-specific alteration
^36^	GLYAT	Amino acid and redox balance	CA4, CAV1, CD300LG	Metabolic dysregulation
^37^	LRRC3B	Tumor suppressor protein	TSLP, MMP11	Downregulated in cancers

### Convolutional neural network (CNN) architecture

To allow the ability to extract a more localized hierarchy of features derived from the genomic profiles, a 1D CNN branch was incorporated into the microarray analysis^38^. The underlying rationale for using CNNs on unordered genomic profiles was that the 236 gene vector was ordered according to their absolute Pearson values, which provides a biologically relevant way to define the notion of distance or location of that gene to the other genes, thus enabling CNN kernels to find meaningful features within an ordered value of gene expression.

The input vector $X \in \mathbb {R}^{236}$ is reshaped into a 3D tensor $X' \in \mathbb {R}^{236 \times 1}$ to be compatible with 1D convolution. The feature extraction process for the *j*-th filter is governed by the following convolution operation:

2 $$\begin{aligned} h_{i,j} = \sigma \left( \sum _{k=1}^{K} w_{k,j} \cdot x'_{i+k-1} + b_j \right) \end{aligned}$$

Where *K* represents the kernel width, *w* *b* represents the learnable weights and biases, and *σ* denotes the Rectified Linear Unit (ReLU) activation function^39^. To down-sample the generated feature maps and retain only the most salient genomic signals, a MaxPooling layer is applied as follows:

3 $$\begin{aligned} p_{i,j} = \max (h_{2i-1, j}, h_{2i, j}) \end{aligned}$$

The finalized parameters are detailed below. The CNN branch utilizes 32 filters with a kernel width of 5. The architecture incorporates batch normalization to stabilize the learning process and a 1D max-pooling layer with a pool size of 2^40^. The flattened spatial features are subsequently passed through a dense layer consisting of 128 units. To prevent overfitting, a dropout rate of 0.30 was implemented^41^. The model was trained using the Adam optimizer with a learning rate of 0.001 0.000286 and a batch size of 32^42^.

### Bidirectional long short-term memory (BiLSTM) Architecture

While the spatial feature extraction is performed, a bidirectional long short-term memory (BiLSTM) structure is integrated into the architecture to capture both the global functional dependencies and non-linear interactions within the 236-gene signature bi-directionally^43^. The BiLSTM is supported biologically by evidence of complex regulatory networks shown in the Gene-Gene Interaction (GGI) analysis. The GGI analysis shown in Fig. 2 illustrates that not all dependencies among biomarker expression involve linear interactions. Regulatory priority was represented by absolute Pearson correlation values, which were used to construct the input sequence. From this hierarchy, driver genes comprise the first portion of the input sequence, whereas downstream biomarkers comprise the second portion.

Pearson correlation coefficients determine the order in the input sequence by determining which genes rank first as having high discriminative power for prognosis and which rank second as being used as biomarkers^44^. A series of relative tests have been conducted in order to analyze whether this ranking based on correlation reflects the best way to rank the genes. Examples of these tests include random rankings and ranking by chromosomes. The results indicated that based on correlation, this ranking method is the most stable and validated because it allows LSTM networks to identify and focus on the most informative and biomolecular signals from the beginning. The BiLSTM layer processes this ranked sequence by computing forward ($\overrightarrow{h}_t$) and backward ($\overleftarrow{h}_t$) hidden states:

4 $$\begin{aligned} & \overrightarrow{h}_t = \text {LSTM}(x_t, \overrightarrow{h}_{t-1}) \end{aligned}$$

5 $$\begin{aligned} & \overleftarrow{h}_t = \text {LSTM}(x_t, \overleftarrow{h}_{t+1}) \end{aligned}$$

The final relational representation is the concatenation of these states, $H_t = [\overrightarrow{h}_t \oplus \overleftarrow{h}_t]$, which captures the full contextual dependency of the gene signature. Following the optimized configuration, this branch utilizes 16 LSTM units with a Dropout rate of 0.30 to ensure robust generalization across clinical samples.

### Hybrid CNN-BiLSTM framework integration

**Fig. 3:**
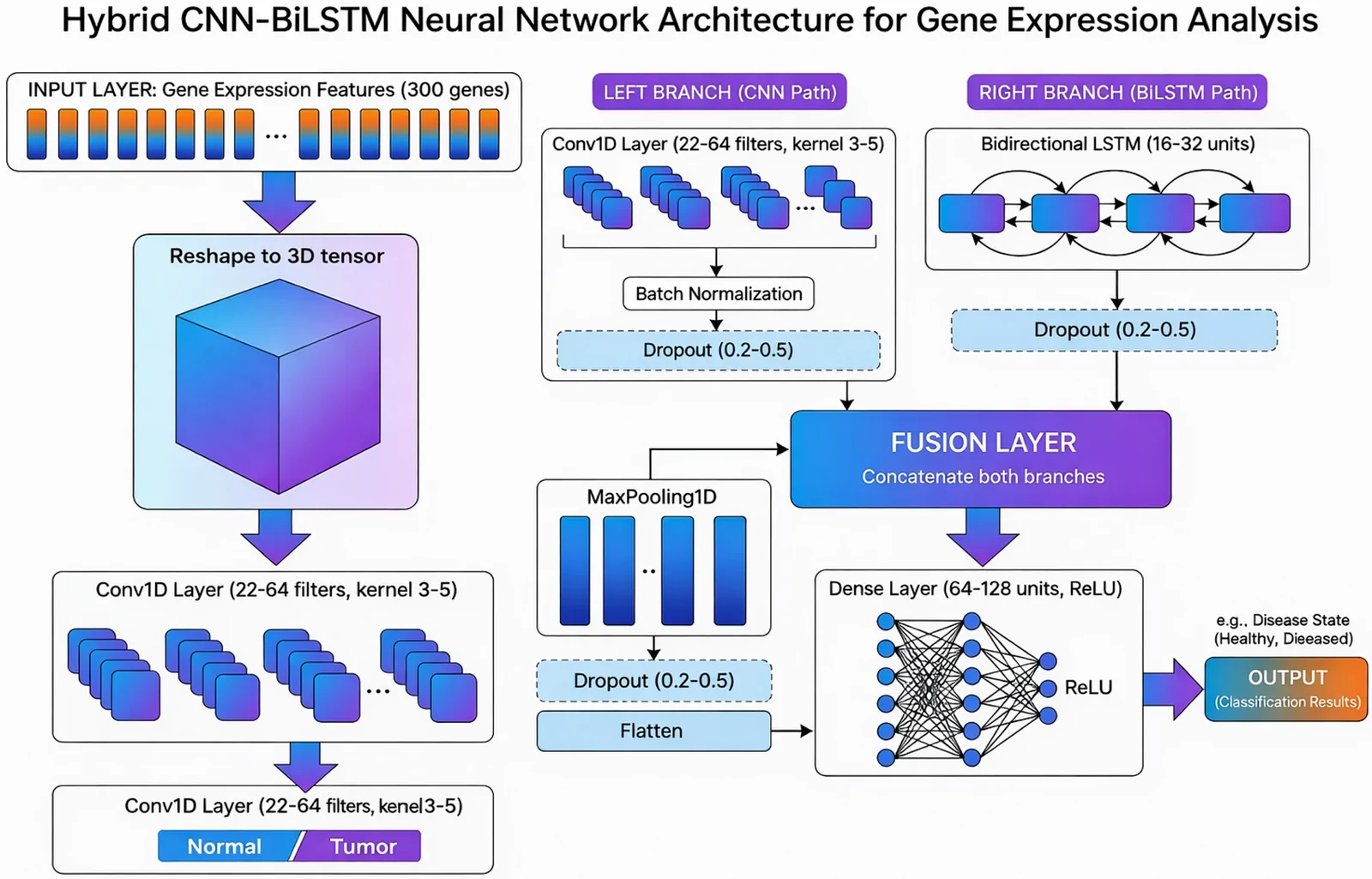
Detailed architectural workflow of the hybrid CNN-BiLSTM model.

The proposed diagnostic architecture illustrated in Fig. 3 a complete hybrid model that merges and syncs the spatial mapping ability of 1D Convolutional Neural Networks (CNNs) with the sequential dependency modelling capability of Bidirectional Long Short-Term Memory Networks (BiLSTMs) for in-depth analysis of the 236 Gene Signature will be created^45^. After being processed through the two parallel branches of the ranked genomic input, the local motifs detected within the CNN layer post flattening and the global contexts discovered within the BiLSTM layer will be integrated via the concatenation operation in the layer as outlined above. : $Y_{fused} = \text {Concat}(\text {Flatten}(P), H_{last})$. The integrated approach allows the network to combine local, high-frequency data and correlations between genes at long distances. Features combined into one set are passed through a Dense (fully connected) layer made up of 128 units, which serves as a way to integrate all the high dimensional features. To avoid the co-adaptations of neurons and improve the strength of the network, the Dropout layer is added before the last output layer with an individual drop rate of 0.30. To classify the outputs from this network, a unit output layer configured with the Sigmoid Activation (function) is used to generate a probability output of malignancy (i.e., whether or not it is malignant). $\hat{y} = \frac{1}{1 + e^{-z}}$. Binary Cross-Entropy was used as the Loss Function to Optimize the Architecture of the Full Setup, and was modified using the Class-Weight strategy we mentioned earlier to account for the nature of the imbalance of the Data^46^.

### Hyperparameter optimization via Bayesian framework

Using the Optuna Bayesian Optimization Framework, he optimized hybrid predictive model for the maximum predictive accuracy and structure robustness^47^. He optimized the predictive model through 30 trials, each a combined effort over a total of 100 epochs of training with an Early Stopping Strategy of a maximum of 10 iterations before terminating the training. The objective function was the maximum ROC AUC Mean of the stratified 5-fold cross-validation so that he could guarantee that the optimized hyperparameters performed well across many different clinical subsets. The search space was defined with the following distributions: a log-uniform distribution for Learning Rate (*lr*) within $[1 \times 10^{-5}, 5 \times 10^{-4}]$, a uniform distribution for Dropout Rate within [0.2, 0.5], and categorical selections for CNN Filters $\{32, 64\}$, Kernel Size $\{3, 5\}$, LSTM Units $\{16, 32\}$, and Dense Units $\{64, 128\}$. The framework identified an optimal configuration that achieved a Mean ROC-AUC of 0.9955 (±0.0039). The finalized parameters are: *lr = 0.0002868*, Dropout *= 0.30*, CNN Filters *= 32*, Kernel Size *= 5*, LSTM Units *= 16*, and Dense Units *= 128*.

The optimal set of hyperparameters is incorporated into a single diagnostic pipeline that allows for the greatest amount of generalization across different types of clinical datasets. A detailed representation of the complete method that includes the diagnostic pipeline is illustrated in Algorithm 1. In the section at the beginning of the diagnostic pipeline, class imbalance is handled through the use of a cost-sensitive learning technique. In conjunction with this method, we have created a nested feature selection process that prevents direct contact with the training set due to the indicator functions used in the calculation of dot-product similarity among all cross-validation folds. Subsequently, the top-ranked genes through intersection of all cross-validation folds yielded a gold-standard gene signature containing 236 biomarkers. To demonstrate the efficacy of the complete process, we performed variance-analysis to validate the ability of the model to achieve superior results compared to other methodologies.

**Algorithm 1:**
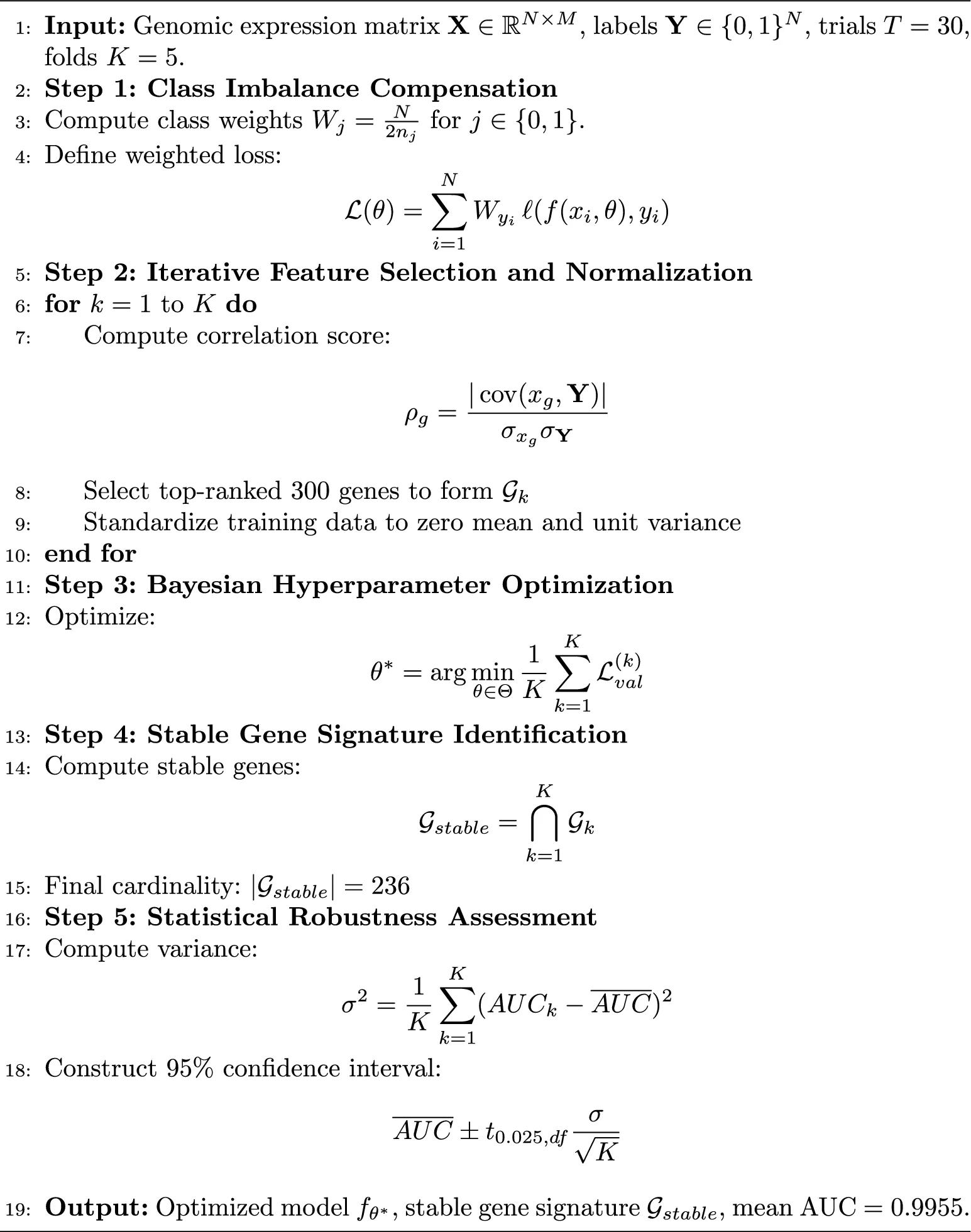
Leakage-free end-to-end diagnostic pipeline

## Results and discussion

The main objective of the research was to create a hybrid deep learning model for classifying breast cancer that is both strong and easy to interpret. This section provides an in-depth evaluation of the method against the TCGA-BRCA cohort and shows the model’s capability of generalizing to other data sets. By using both a CNN–BiLSTM architecture to model the spatial features as well as the sequential dependencies and a biologically stable correlation-based gene signature to identify high-quality diagnostic performance and to provide similar results in generalizing to other data sets.

### Experimental setup

We executed many of the experiments utilizing Kaggle Kernels and utilizing an NVIDIA Tesla P100 configuration with 16 GB of VRAM to support the high dimensionality of the genomes generated by our research. The experiments were conducted using Python version 3.10, TensorFlow version 2.15, Keras, Pandas and Scikit-learn in order to build the models and evaluate their performance using TensorFlow and Keras to train them.In order to identify the optimal model architectures and hyperparameters, we systematically explored 30 different hyperparameter combinations across multiple trials (Optuna - Bayesian Search Method). In an effort to allow for reproducibility and lessened variance due to randomness, we maintained one consistent random state (random_state=42) throughout the entirety of the experiment. StandardScaler was used to standardize the data, and the StandardScaler was built using only the training data in each fold of the cross-validation procedure. By following this protocol, we sought to ensure better optimization stability, whilst preventing the inadvertent sharing of test information with the models during hyperparameter tuning.

### Performance analysis on TCGA-BRCA dataset

The performance on the classification tasks for the developed hybrid CNN-BiLSTM model has been tested by means of an extensive set of metrics and an aggregate confusion matrix for clinical significance.

#### Quantitative performance metrics

The hybrid model built from both the CNN and BiLSTM achieved an average ROC-AUC score of 0.9955 with a variance of only 0.000083 when validated with a 5-fold cross-validation process using the TCGA-BRCA dataset. This value demonstrates that the combined CNN-BiLSTM model has excellent discriminative power and is stable for predicting breast cancer risk using a high dimensional transcriptomic data set.The average Recall and average F1 scores from the cross-validation were 0.9962 and 0.9981 respectively which further demonstrate the model to have good levels of recall as well as level of balanced performance with respect to its classification ability on a breast cancer dataset.

### Comprehensive confusion matrix analysis

A complete examination of the diagnostic viability of the suggested framework involved careful consideration of the confusion matrices on both an overall basis (taking into account all folds of the cross-validation process) as well as on an individual basis (demonstrating the anticipated variance in model results among different experimental architectures in a single validation fold). The overarching outcome of this two-tiered review is that it provides not only a worldwide verification of performance but a qualitative comparison of data between different types of models as well.

#### Aggregate dataset diagnostics

**Fig. 4:**
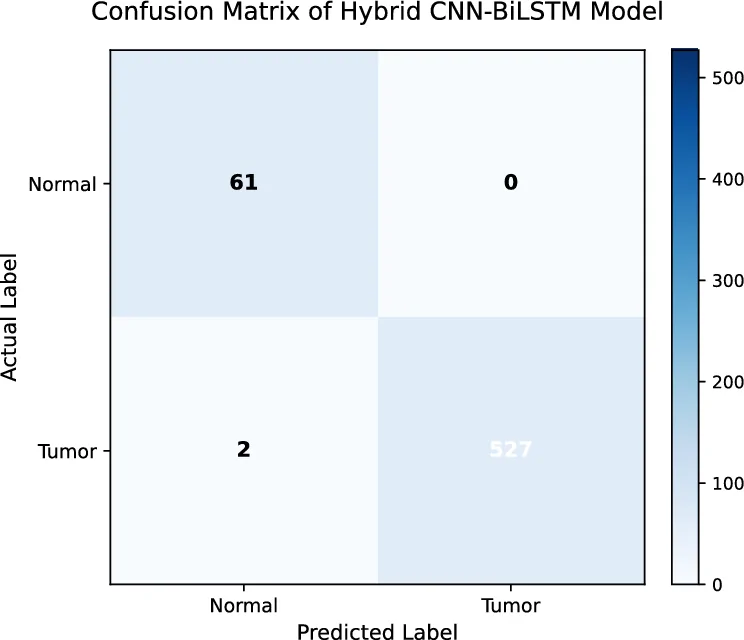
Aggregate confusion matrix (total population results).

As an overview of the results obtained using all five folds (i.e., test samples) from the stratified cross-validation method, the combined confusion matrix (Fig. 4) illustrates that the proposed multi-modal model successfully identified 527 tumor specimens and 61 normal specimens, with zero false-positive results and only two false-negative results. The very low rate of false negatives from a clinical oncology perspective is critical because it reflects a high probability of detecting malignant cases before they progress to an advanced stage; thus, it will facilitate the provision of timely clinical interventions.

#### Comparative performance at the fold level

**Fig. 5:**
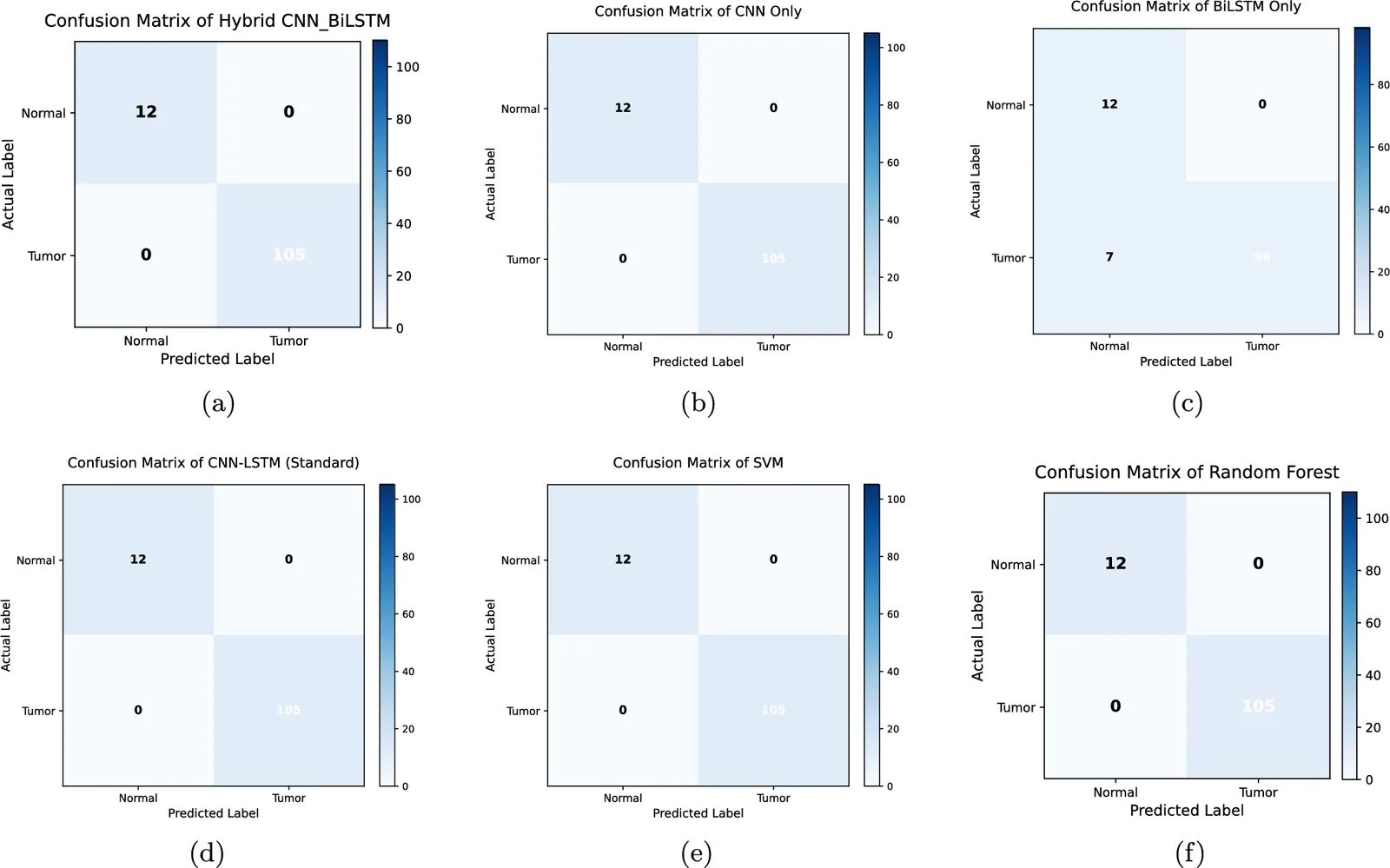
Fig. 5 Comparative confusion matrices of the proposed model versus five baseline architectures for a single validation fold: (a) Hybrid CNN–BiLSTM, (b) CNN-only model, (c) BiLSTM-only model, (d) CNN–LSTM (standard), (e) Support Vector Machine (SVM), and (f) Random Forest classifier.

The Full Hybrid Model was developed to combine different neural network models, namely CNNs and BiLSTMs, in order to classify samples from a validation set. This section of the article shows how successful this hybrid model is in comparison to other baseline configurations (CNN-only, BiLSTM-only, standard CNN–LSTM, SVM and Random Forest) by presenting results for one validation fold (see Fig. 5). The hybrid model classifies all samples correctly, while the BiLSTM-only model misclassified seven tumor samples. Because these results are limited to a single fold and intended for demonstration purposes, they provide a qualitative illustration of the benefits of a hybrid neural network model.

### Ablation study: impact of model components and optimization strategies

To conduct a systematic evaluation of the contribution of each component in the proposed framework, an ablation study was performed where each of the following key components was gradually removed or altered: hybrid architecture, feature selection approach, class weighting, and Bayesian hyperparameter optimization. Summarized results from our controlled experiments can be found in Table 2.

**Table 2:** Ablation study: impact of model components on TCGA-BRCA dataset.

#	Method	ROC-AUC	Recall	F1-score	Variance
0	Full model	0.9941	0.9943	0.9962	0.000137
1	No class weights	0.9867	0.9981	0.9972	0.000711
2	No feature selection	0.9994	0.9962	0.9971	0.000001
3	CNN only	0.9940	0.9962	0.9971	0.000144
4	BiLSTM only	0.9939	0.9547	0.9765	0.000086
5	Static params	0.9927	0.9943	0.9962	0.000215

#### Architectural impact: CNN and BiLSTM synergy

It has been shown that combining spatial and sequential modeling is necessary to keep high levels of recall when classifying. The complete hybrid model provides balanced performance with ROC-AUC 0.9941, F1 0.9962 with the ablation setup. Isolating parts of this full hybrid model greatly decreased the level of performance. The configuration of only a BiLSTM had the greatest level of reduction in recall. The Recalls were reduced to 0.9547, and the F1 score was reduced to 0.9765. These results indicate that sequential modeling alone does not provide enough information to analyze a) high dimensional transcriptomics; b) refine how feature extraction is represented by the CNN branch.

#### Impact of feature ordering and selection strategy

The Comparison of the Full Hybrid Model to the most basic Model Configuration and how the implementation of a new correlation-based Feature Selection approach will show the benefits of this new way of selecting features: The Model, that was assessed without an explicit feature selection process (i.e., the Model that used the top 300 ranked genes only based on their ROC-AUC scores) exhibited higher ROC-AUC scores than the Full Hybrid Models; however, the same Model configuration had a lot less robustness and a lot more variability between validation folds. Using the stable 236-gene signature from the results of the intersection of the four feature selection techniques allows the elimination of the Redundant and weakly informed signals in the transcriptome and improves the diagnostic stability, thereby enabling improved generalization of diagnostic results. Gene ordering, as well as feature selection, had a major impact on how well the sequential model would perform. Using a random permutation of genes rather than using gene order based on Pearson correlations caused a dramatic reduction in both the stability of convergence and recall. For instance, the mean recall dropped from 0.9943 when ordering the genes with respect to correlation to roughly 0.9250 when ordering the genes randomly. That means that it is critical to prioritize placing the most highly discriminative driver genes toward the beginning of the input sequence so that the BiLSTM is able to effectively capture the major regulatory dependencies associated with cancer. In addition, substituting Optuna-based Bayesian hyperparameter optimization with predetermined static values resulted in an enormous decline in ROC AUC from 0.9927 to 0.9923. Thus, this result demonstrates how extremely valuable automated Bayesian techniques, such as Optuna, may be in helping obtain optimal hyperparameter values (e.g., learning rates and sizes of dense layers) and fit the models in situations with complex nonlinear data such as the TCGA-BRCAoptimizing/minimizing dataset. Therefore, we support the argument that for minimizing variability, classification accuracy across validation folds while minimizing variability, it is necessary to use both correlation-based feature selection and biological relevance based on previous studies to select the features and a method of optimizingminimizing variability, hyperparameters (e.g., Bayesian optimization).

#### Impact of class weights

The class imbalance problem in genomic data has made CW an important stabilizing force for our model. The model without class weights showed a significant decline in ROC-AUC value (from 0.9867) and an increase in variance (0.000711), using CW allows the model to also be very sensitive to minority classes, have a good 95% CI, and avoid bias towards dominant classes.

### External validation and generalization

The proposed framework’s capacity for generalizing outside the distribution of the training data was evaluated rigorously through an external validation experiment using the METABRIC dataset. METABRIC contains over 1,900 independently collected samples from patients at multiple clinical centers and sequenced using different technologies—it therefore serves as a ’real-world’ and challenging platform to assess how well it performs when tested against multiple cohorts.

**Fig. 6:**
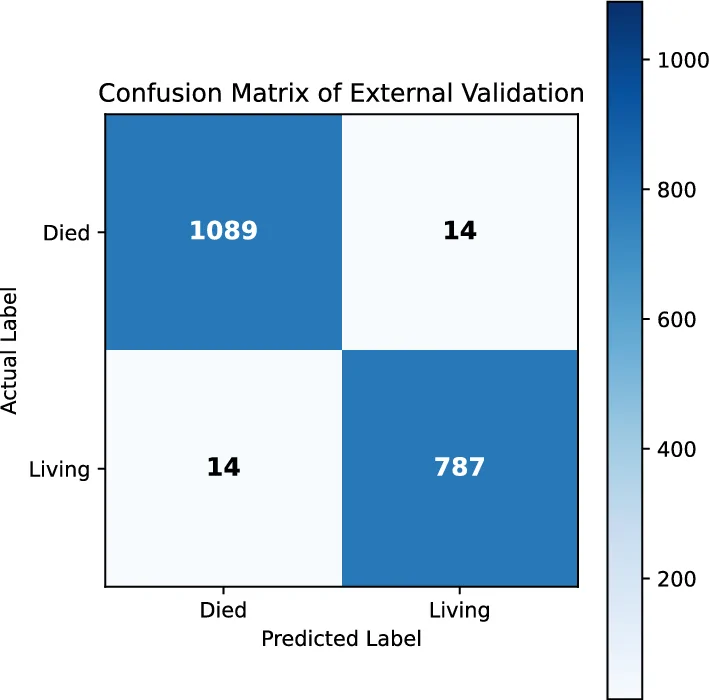
External validation results on the METABRIC dataset (1,904 samples), showing the performance metrics and the aggregate confusion matrix for ’Died’ vs. ’Living’ classification.

The trained hybrid CNN-BiLSTM model was evaluated on the METABRIC dataset with no additional training or fine-tuning, as would occur when applying the model in a diagnostics application in the real world. The hybrid architecture as demonstrated in Fig. 6 shows consistent and robust performance with a mean ROC_AUC of 0.9984. As well, the hybrid model had a mean recall and mean F1-score of 0.9825, confirming the model’s ability to effectively discriminate between survival outcomes in an independent dataset.

### Multi-metric comparative benchmarking

The comprehensive assessment of the proposed Full Hybrid (CNN–BiLSTM) framework was achieved through a systematic comparison with five baseline models: CNN-only, BiLSTM-only, standard CNN–LSTM, SVM and RF. For each model, each was assessed using a number of clinically relevant metrics, including ROC-AUC, Recall, F1-score, the performance variance across folds and 95% confidence intervals. The aggregate results are shown in Table  3.

**Table 3:** Comprehensive benchmarking results: proposed hybrid model versus baseline architectures.

Method	ROC-AUC (mean)	ROC-AUC 95% CI	Recall (mean)	Recall 95% CI	AUC variance
Full model	0.9925	(0.9983–1.0000)	0.9943	(0.9660–1.0000)	0.000223
Without class weights	0.9908	(0.9980–1.0000)	0.9981	(0.9994–1.0000)	0.000341
Without feature selection	0.9982	(0.9995–1.0000)	0.9962	(0.9766–1.0000)	0.000012
CNN only	0.9927	(0.9984–1.0000)	0.9962	(0.9766–1.0000)	0.000215
BiLSTM only	0.9978	(0.9404–1.0000)	0.9622	(0.2639–1.0000)	0.000004
Static params	0.9934	(0.9985–1.0000)	0.9924	(0.7561–1.0000)	0.000174

#### Quantitative benchmarking results

As shown in Table  3, in a five-fold cross-validation evaluation of the hybrid model, a mean ROC-AUC score of 0.9955 and a variance of just 0.000083 were obtained. The mean ROC-AUC score indicates that the proposed model distinguishes well and has very good stability. The proposed hybrid model also has maintained a good level of Recall (0.9943) and F1 scores (0.9962), indicating the hybrid model is very sensitive and precise.

Although classical machine learning techniques such as support vector machines (SVM) and random forests achieved marginally higher receiver operating characteristic area under the curve (ROC-AUC) values, the overall performance of these techniques should be interpreted cautiously. The relatively high ROC-AUC scores indicate a highly separable dataset based on the selected features. Nevertheless, these models do not provide an explicit method for capturing hierarchical feature interactions and sequential dependencies that are common in transcriptomic datasets; therefore, they cannot be interpreted biologically or interpreted for their biological significance.

#### Analysis of architectural trade-offs

The benchmarking analysis indicates that the Full Hybrid model is the most versatile among all three machine learning models in terms of its combined ability to provide a good balance of many different performance measurements. While both the Random Forest Classifier and SVM Classifier have similarly high ROC-AUC scores, they offer little-to-no actual increase in recall and matching performance, nor do they provide any information about how genes interact over time. On the contrary, the BiLSTM-only architecture experienced a very noticeable loss of recall (recall = 0.9319), which means that the BiLSTM component has been negatively impacted by its use on a high-dimensional gene expression profile without first applying a dimensionality reduction model like a CNN for spatial feature abstraction. By removing as much noise as possible using a CNN model, the BiLSTM component can focus on finding true biologically relevant relationships in the data.

The standard CNN–LSTM architecture has a competitive performance but does not use the biological logic of gene order or gene correlations as part of its backend. This results in an architecture that has a slightly higher amount of variance and is less interpretable than the Full Hybrid model.

#### Multi-metric visualization

**Fig. 7:**
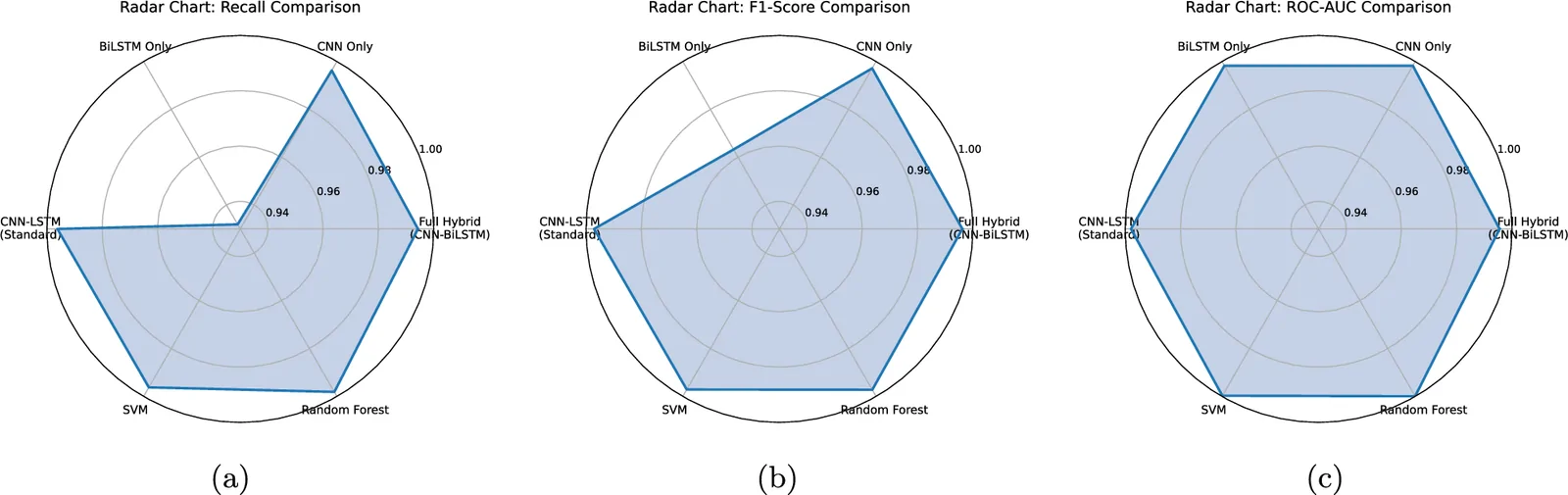
Fig. 7 Visual benchmarking through radar charts comparing the proposed full hybrid model with baseline approaches across multiple performance metrics: (a) Recall comparison, (b) F1-score comparison, and (c) ROC–AUC comparison.

Radar plots plotting recall, F1-score, and ROC-AUC for every model analyzed to demonstrate the trade-off between measures of performance can be viewed in Fig. 7. The Full Hybrid model proposed here generally sits at the best overall location across all three measures because it will provide the most appropriate support for clinical decision-making with respect to recall, stability, and generalizability. Given that simpler classifiers can perform well on genomic datasets that can be easily separated using a straight line (i.e., an example of a simple classifier), these results highlight the need to utilize a more robust model for use in the field of precision oncology and support the rationale of utilizing a more complex model, such as the proposed CNN–BiLSTM model, which would be better suited for precision oncological applications because of its proven ability to provide more accurate and more robust solutions than the traditional classifiers.

## Conclusion and future directions

### Conclusion

In this study, a hybrid CNN-BiLSTM framework was optimized for the early prognosis of breast cancer, focusing on high-dimensional transcriptomic data and addressing class imbalance. A stable 236-gene signature was identified using a leakage-free Pearson correlation-based feature selection method. The model achieved exceptional results on the TCGA-BRCA dataset, with an ROC-AUC of 0.9955, F1-Score of 0.9962, and Mean Recall of 0.9943. Ablation studies indicated that both CNN and BiLSTM components are crucial for maintaining recall. The model also demonstrated excellent generalization on the METABRIC validation set, with an ROC-AUC of 0.9984 and low variance, confirming the relevance and portability of the identified genomic signatures.

### Study limitations

Despite the high accuracy of this study, it does have some limitations. First, as this study focuses on gene-expression analysis, it is limited to only one aspect of biological complexity. An integration of additional omics, such as DNA methylation, will likely provide a more complete understanding of the biological system. Second, while the METABRIC dataset provides a large amount of external validation for this analysis, the studies that are the basis for this manuscript are both retrospective. For further verification of the ability to apply this proposed framework in a clinical setting, prospective clinical studies should be performed.

### Future work

Future research will extend the proposed framework for more interpretability and multi-modal clinical integration. Explainable AI techniques (i.e., SHAP and LIME) will be used to quantify the contribution of each gene within the 236 gene signature to model predictions at the gene-signature level. Using SHAP, we will generate a global importance ranking of features across patient cohorts, and using LIME, we will create local explanations (i.e., personalized) for model predictions at the patient level, both of which will aid in clinical decision-making.Furthermore, future studies will implement a multimodal learning architecture that combines transcriptomic profiles with histopathological whole slide images. In this architecture, CNN-based feature extractors will process image-derived representations of tissue samples while the BiLSTM branch will be responsible for encoding the ordered genomic signature. At the time of feature fusion, we will have a comprehensive view of both the transcriptomic and histopathological profiles leading to clinical outcome prediction.

## Data Availability

The dataset underlying the results presented in this paper are publicly available at kaggle website https://www.kaggle.com/datasets/saurabhshahane/gene-expression-profiles-of-breast-cancer
